# *Mycoplasma genitalium* Incidence, Coinfection, and Antibiotic Resistance: A Prospective Study at a Walk-In Clinic in Los Angeles County, CA

**DOI:** 10.1093/ofid/ofae419

**Published:** 2024-07-18

**Authors:** Adam Carl Sukhija-Cohen, Henna Patani, Antigone Contessa Robinson, Matthew Ramos Santos, Yancy Granados

**Affiliations:** Palo Alto Medical Foundation Research Institute, Center for Health Systems Research, Sutter Health, Palo Alto, California, USA; Public Health Division, AIDS Healthcare Foundation, Los Angeles, California, USA; Public Health Division, AIDS Healthcare Foundation, Los Angeles, California, USA; Public Health Division, AIDS Healthcare Foundation, Los Angeles, California, USA; Public Health Division, AIDS Healthcare Foundation, Los Angeles, California, USA

**Keywords:** antibiotic resistance, coinfections, community clinic, mycoplasma genitalium, sexually transmitted infections

## Abstract

Among 98 participants with penile discharge symptoms of *Chlamydia trachomatis* or *Neisseria gonorrhoeae* at a walk-in sexual health clinic, 11 were diagnosed with *Mycoplasma genitalium*, 10 had antibiotic resistance, and 6 were incorrectly presumptively treated. Our findings highlight the importance of public health strategies and research to curb *M genitalium*.

*Mycoplasma genitalium* is a bacterial sexually transmitted infection (STI) first reported in 1981 [1]. It is estimated to be less common than *Chlamydia trachomatis* (chlamydia) and more common than *Neisseria gonorrhoeae* (gonorrhea) in the United States. In other words, there were between 648 056 and 1 649 716 *M genitalium* infections in 2022 [2, 3]. *Mycoplasma genitalium* is a major cause of urethritis among men; cervicitis, pelvic inflammatory disease, preterm delivery, spontaneous abortion, and infertility among women; and is associated with HIV for men and women [4].

The first test authorized for *M genitalium* by the U.S. Food and Drug Administration (FDA) was the Aptima *M genitalium* nucleic acid amplification test by Hologic (Marlborough, MA) in 2019 [5]. However, there is still limited national surveillance [6] and prevalence estimates vary widely [7, 8]. *Mycoplasma genitalium* has also shown resistance to macrolides and fluoroquinolones, which is why the recommended treatment for all cases is doxycycline followed by either azithromycin or moxifloxacin [4]. There is currently no FDA-approved resistance test for *M genitalium*.

Symptoms of *M genitalium* infection may include vaginal and penile discharge as well as burning sensation when urinating, which are similar to symptoms of chlamydia/gonorrhea infection [9]. The Centers for Disease Control and Prevention (CDC) STI treatment guidelines recommend presumptive chlamydia/gonorrhea treatment if patients present symptoms of chlamydia/gonorrhea infection [4]; however, incorrect presumptive treatment still occurs and patients may instead have *M genitalium*, in which chlamydia/gonorrhea treatment would be insufficient. Beginning in 2021, the CDC recommended testing for *M genitalium* for men with recurrent nongonococcal urethritis and women with recurrent cervicitis [10].

AIDS Healthcare Foundation (AHF) operates free walk-in sexual health clinics in the United States for patients seeking testing and treatment for chlamydia, gonorrhea, syphilis, and HIV. AHF's second largest STI clinic, Wellness on Western, is in Los Angeles County, California, and predominantly serves men who engage in high-risk sexual behaviors. The goal of this study is to better understand the incidence of *M genitalium* among male patients and their demographics, risk behaviors, coinfections, and antibiotic resistance.

## METHODS

Using a prospective observational study design, adult male patients who presented penile discharge symptoms of chlamydia/gonorrhea at AHF's Wellness on Western were recruited to participate. Enrollment began in March 2023 and ended in August 2023. Participants provided a first-catch urine sample into a urine collection cup for standard-of-care chlamydia/gonorrhea testing. Participants were then presumptively treated for chlamydia/gonorrhea. Research staff aliquoted 2 mL of the collected urine into an Aptima urine transport container for Laboratory Corporation of America Holdings (Labcorp; Burlington, NC) to conduct testing using the FDA-approved Aptima assay. Research staff then aliquoted 2 mL of urine into a second Aptima urine transport container to store in Wellness on Western's on-site −80 °C laboratory freezer for later antibiotic resistance testing at the University of Alabama at Birmingham Diagnostic Mycoplasma Laboratory. If a participant tested negative for *M genitalium*, their freezer-stored urine sample was disposed of. If a participant tested reactive, University of Alabama at Birmingham conducted molecular detection of both macrolide and fluoroquinolone resistance.

Participants with confirmed *M genitalium* infection were contacted up to 3 times by research staff to return to Wellness on Western for treatment. Participants who started their treatment were then contacted 2 weeks later to confirm that treatment was completed and that symptoms were no longer present.

Participant demographics, risk behaviors, HIV status, and STI test results were all collected via electronic medical records. All analyses were performed using Microsoft SQL, Excel, and SAS 9.4. This study was approved by the Advarra institutional review board.

## RESULTS

Table 1 provides a summary of the demographics for both the 98 male participants who enrolled in the study and the 11 (11.2%) who tested reactive for *M genitalium*. The table also includes selected risk behaviors for the 11 participants diagnosed with *M genitalium*. Three were living with diagnosed HIV. Five self-reported having condomless sex in the past 12 months. All 11 participants self-reported having multiple sexual partners: 3 (27.3%) had 2–9 sexual partners, another 3 (27.3%) had 10–19 sexual partners, and 2 (18.2%) had 40 or more sexual partners. Five of the 11 participants (45.5%) diagnosed with *M genitalium* had coinfections with other STIs: 4 (36.4%) with gonorrhea only and 1 (9.1%) with both chlamydia and syphilis. All 3 participants with diagnosed HIV had coinfections with other STIs (not summarized in the table). When tested for resistance, 9 (81.8%) had macrolide resistance and 1 (9.1%) had both macrolide and fluoroquinolone (in this case, ParC) resistance. None of the 11 cases reported GyrA fluoroquinolone resistance.

**Table 1.** Demographics and Risk Factors by *Mycoplasma genitalium* Test Results Among Male Patients Presenting Penile Discharge Symptoms at AIDS Healthcare Foundation's Wellness on Western Walk-in Sexual Health Clinic in Los Angeles County, CA, March–August 2023

	*Mycoplasma genitalium* test results		
	Negative	Reactive	Total
	N (column %)	N (column %)	N (column %)
Age group, years			
18–24	2 (2.3)	1 (9.1)	3 (3.1)
25–29	23 (26.4)	2 (18.2)	25 (25.5)
30–34	25 (28.7)	4 (36.4)	29 (29.6)
35–39	21 (24.1)	2 (18.2)	23 (23.5)
40+	16 (18.4)	2 (18.2)	18 (18.4)
Race			
Asian	4 (4.6)	0 (0.0)	4 (4.1)
Black/African American	18 (20.7)	5 (45.5)	23 (23.5)
Native Hawaiian/Pacific Islander	2 (2.3)	0 (0.0)	2 (2.0)
White	28 (32.2)	4 (36.4)	32 (32.7)
Other/refused	35 (40.2)	2 (18.2)	37 (37.8)
Ethnicity			
Hispanic/Latino	37 (42.5)	3 (27.3)	40 (40.8)
Not Hispanic/Latino	43 (49.4)	6 (54.5)	49 (50.0)
Other/refused	7 (8.0)	2 (18.2)	9 (9.2)
Sexual orientation			
Gay	44 (50.6)	7 (63.6)	51 (52.0)
Bisexual	16 (18.4)	1 (9.1)	17 (17.3)
Heterosexual	19 (21.8)	2 (18.2)	21 (21.4)
Other/refused	8 (9.2)	1 (9.1)	9 (9.2)
HIV status			
Living with HIV	-	3 (27.3)	-
Not living with HIV	-	8 (72.7)	-
Condomless sex in the past 12 months			
Condomless sex	-	5 (45.5)	-
No condomless sex	-	0 (0.0)	-
Unknown/refused	-	6 (54.5)	-
Number of sexual partners			
0–1	-	0 (0.0)	-
2–9	-	3 (27.3)	-
10–19	-	3 (27.3)	-
20–29	-	0 (0.0)	-
30–39	-	0 (0.0)	-
40+	-	2 (18.2)	-
Unknown/refused	-	3 (27.3)	-
STI coinfection			
No coinfection	-	6 (54.5)	-
Gonorrhea only	-	4 (36.4)	-
Chlamydia and syphilis	-	1 (9.1)	-
Antibiotic resistance			-
No resistance	-	1 (9.1)	-
Macrolide -23S rRNA	-	9 (81.8)	-
Fluoroquinolone-parC	-	0 (0.0)	-
Fluoroquinolone-gyrA	-	0 (0.0)	-
Macrolide -23S rRNA and fluoroquinolone-parC	-	1 (9.1)	-
Total (row %)	87 (88.8)	11 (11.2)	98 (100.0)

## DISCUSSION

This study documents 11 *M genitalium* infections among 98 adult male patients (11.2%) presenting penile discharge symptoms of chlamydia/gonorrhea. Six of the 11 participants were presumptively treated for chlamydia/gonorrhea despite ultimately testing negative for both. All but 1 of the 11 *M genitalium* cases were resistant to macrolides, fluoroquinolones, or both. Our findings highlight the challenges of presumptive treatment and the risk of antibiotic resistance.

There are 4 important limitations in this study. First, participants were enrolled only after a medical provider confirmed a patient presented penile discharge as a symptom of chlamydia/gonorrhea and would receive presumptive treatment for chlamydia/gonorrhea. Most patients seeking STI services at AHF's Wellness on Western do not present symptoms and were therefore not invited to participate in this study. Second, only male patients were recruited because the clinic does not offer vaginal swabs for women as standard-of-care and vaginal swabs are the preferred sample for *M genitalium* testing. It is likely more cases would have been detected had asymptomatic patients and female patients been recruited. Third and fourth, participants were recruited from only 1 clinic in Los Angeles County and the sample size was small, both of which limit the generalizability of our findings.

Leandro Mena, director of the CDC's Division of STD Prevention, said in 2023, “The U.S. STI epidemic shows no signs of slowing” [11], and STI funding has declined for the past two decades [12]. *Mycoplasma genitalium* may be contributing to incorrect diagnoses and treatments of reportable STIs like chlamydia and gonorrhea. In addition, the high proportion of antibiotic resistance found in this study as well as others demonstrates the importance of continued public health strategies and research to curb the spread of *M genitalium* [13].

## Notes

***Acknowledgments.*** The authors thank Sherrelle Banks, Karla Quiteno, Gjermayne Wilson, Gerald Ramirez, Jesus Gonzalez, Ellie Cohen, and Anisha Bhargava for their help in study implementation, data collection, and data extraction.

***Author contributions.*** A. C. S. and H. P. conceptualized the project. H. P., M. R. S., and Y. G. developed data collection instruments and performed data management. M. R. S completed all institutional review board and ethical documentation needed. Y. G. recruited participants and oversaw research associates. H. P. and A. C. R. conducted descriptive statistics. A. C. S. wrote the first draft of the manuscript. All authors provided substantive input into the final manuscript.

***Data availability.*** Data not publicly available.

***Patient consent statement*.** Patient's written consent was obtained.

***Financial support*.** The authors report no funding sources.
